# Effectiveness of Adult Health Promotion Interventions Delivered Through Professional Sport: Systematic Review and Meta-Analysis

**DOI:** 10.1007/s40279-022-01705-z

**Published:** 2022-06-16

**Authors:** Emma S. George, Aymen El Masri, Dominika Kwasnicka, Alannah Romeo, Sarah Cavallin, Andrew Bennie, Gregory S. Kolt, Justin M. Guagliano

**Affiliations:** 1 School of Health Sciences, Western Sydney University, Sydney, NSW Australia; 2 Translational Health Research Institute (THRI), Western Sydney University, Sydney, NSW Australia; 3 SWPS University of Social Sciences and Humanities, Wroclaw, Poland; 4 NHMRC CRE in Digital Technology to Transform Chronic Disease Outcomes, School of Population and Global Health, University of Melbourne, Melbourne, VIC Australia

## Abstract

**Background:**

Researchers are capitalising on the strong connections that sport fans have with their teams for health promotion programmes, yet no existing systematic reviews have evaluated the effectiveness of interventions delivered through professional sport.

**Objective:**

The aim of this study was to systematically collate, evaluate, and synthesise the evidence on health promotion interventions implemented in professional sport settings.

**Methods:**

Randomised controlled trials reporting on adult health promotion initiatives delivered in professional sport settings were identified through electronic database searches in CINAHL, MEDLINE, SPORTDiscus, Scopus, the Cochrane Central Register of Controlled Trials (CENTRAL), and Google Scholar. Data on health-related outcomes (e.g., weight, physical activity, dietary intake) were extracted and synthesised, and random effects meta-analyses were conducted to examine effects for weight and waist circumference. Risk of bias was examined using the Cochrane risk-of-bias tool for randomised controlled trials (RoB 2).

**Results:**

Six studies reporting on five unique interventions met the inclusion criteria, and all included studies were gender-sensitised and exclusively targeted men. Intervention effects were observed for several health outcomes, including physical activity, dietary intake, and psychosocial health. All studies aimed to reduce weight, and for most studies (*n* = 4), weight was a primary outcome, either of the included study or to inform a future definitive trial. Findings from the meta-analysis revealed an overall significant difference in change in weight of − 3.2 kg (95% confidence interval [CI] − 4.6 to − 1.8) and waist circumference of − 3.9 cm (95% CI − 4.9 to − 2.8), both in favour of the intervention group at 12 weeks. Intervention effects were also reported for several other health outcomes (e.g., physical activity, dietary intake, psychosocial health); however, they were not consistently measured across the studies and thus were not meta-analysed.

**Conclusion:**

Health promotion interventions delivered through professional sporting organisations can significantly improve weight- and lifestyle-related health outcomes. Representation across the socioeconomic spectrum and across culturally and linguistically diverse groups was limited. As only a limited number of studies met the inclusion criteria for this review, a need exists for rigorously designed interventions, standardised intervention approaches, with long-term follow-up, and the potential for scalability.

**PROSPERO Registration Number:**

CRD42019123295.

**Supplementary Information:**

The online version contains supplementary material available at 10.1007/s40279-022-01705-z.

## Key Points

**Table:** 

Professional sport can be used as a vehicle to promote health, leading to significant improvements in a range of physical and psychosocial health outcomes in men.
Only six studies met the inclusion criteria, highlighting a need for more rigorously designed interventions across diverse sports, and geographic regions.
Future interventions should explore opportunities to recruit more culturally and socioeconomically diverse samples.

## Introduction

Noncommunicable diseases, including cardiovascular diseases, cancers, chronic respiratory diseases, and diabetes, contribute to approximately 71% of deaths globally and are of significant public health concern [1]. Many of the risk factors associated with these diseases are modifiable and include overweight and obesity, tobacco smoking, inadequate dietary intake, alcohol consumption, high blood pressure, and physical inactivity [1]. Individual-level interventions provide important opportunities to modify health behaviours such as these; however some of the most potent drivers of poor health are complex and multilevel, influenced by structural, environmental, or political factors, often beyond an individual’s control [2]. These factors disproportionately impact disadvantaged, disenfranchised, and marginalised communities, often leading to inequitable opportunities to achieve good health [3]. It is therefore important to develop health promotion interventions with these factors in mind.

Professional sporting organisations are in an optimal position to affect health positively at various levels. By using their reach and standing within the community, they have the potential to engage their supporters in meaningful ways that extend beyond match attendance and fan identification. Sport fans form strong social and psychological connections to their sports team [4] and the feelings of identity, belonging, and loyalty associated with being a fan have been shown to enhance mental health outcomes and promote social inclusion and connectedness [5]. Many professional sports organisations also have a strong sense of corporate social responsibility to their fans and broader communities they serve [6], and such organisations are increasingly engaging fans for the promotion of physical and mental wellness via community engagement initiatives [7]. Although fan affiliation is a powerful drawcard, until recently this has been an underutilised entry point for rigorously designed and evaluated evidence-based health promotion initiatives, therefore the overall effectiveness of health promotion initiatives in this context was largely unknown [8].

In recent years, the number of studies designed to recruit and engage sport fans for the promotion of physical and mental health has increased. Large-scale programs such as Premier League Health, offered through the English Premier League [9] and Football Fans in Training (FFIT), delivered via the Scottish Professional Football League (SPFL) [8, 10], have successfully capitalised on men’s passion for and affiliation with football as an entry point for health promotion, resulting in improved health and wellbeing outcomes. These interventions have successfully engaged hard-to-reach population groups, including middle-aged males and individuals from low socioeconomic communities, demonstrating that professional sporting organisations are efficacious settings for health promotion. FFIT [8] was the first public health intervention of its kind to be evaluated using a randomised controlled trial (RCT) study design and set an important precedent for health promotion initiatives in professional sport. This study recruited overweight male football fans into a gender-sensitised weight loss and healthy living programme, delivered through 13 SPFL clubs. Gender-tailored intervention designs aligning with the preferences, interests and needs of men have been shown to support weight loss [11, 12] and physical activity [13] in a variety of contexts. The FFIT programme involved face-to-face, group-based education and physical activity sessions held weekly at each club’s home stadium and was gender-sensitised in content, context, and delivery style, which involved peer-supported learning [8, 10]. Harnessing the cultural and masculine appeal of sport, the success of this programme has led to the FFIT model being replicated in football in Europe [14] and Germany [15], ice hockey in Canada [16, 17], rugby union in New Zealand [18], and Australian Rules Football in Australia [19], with further interventions underway and under development.

Several systematic reviews on health promotion in the broader sporting context have been published. For example, Priest et al. have conducted reviews exploring health promotion policies in sporting clubs [20] and examining interventions designed to increase participation in sport [21]. Walzel et al. conducted an integrative review on corporate social responsibility in professional team sport organisations [6], and Curran et al. have examined the role of professional football clubs in promoting mental health [22]. There are however no current systematic reviews examining RCT study designs of health promotion interventions targeting adults, delivered through professional sporting organisations.

The aim of this study was to systematically collate, evaluate and synthesise the evidence on health promotion interventions delivered through professional sporting organisations. Through a qualitative synthesis and meta-analysis, this systematic review provides evidence on the nature and effectiveness of health promotion interventions implemented in a professional or semi-professional sporting context and may help inform the design of future initiatives intended to engage communities through professional sport, and those targeting hard-to-reach or underserved population groups.

## Methods

This systematic review followed the Preferred Reporting Items for Systematic Reviews and Meta-Analyses (PRISMA) guidelines (electronic supplementary material [ESM] Appendix S1) and was prospectively registered in the International Prospective Register of Systematic Reviews (PROSPERO; registration number CRD42019123295).

### Inclusion Criteria

Studies were eligible for inclusion if they met the following criteria:

*Participants:* Studies targeting adults aged 18 years and over who were not professional athletes.

*Intervention:* Interventions targeting any health-related behaviours and outcomes (e.g., weight, physical activity, diet, mental health) that were delivered via a professional or semi-professional sporting organisation were considered.

*Comparison:* Studies must have compared an intervention to a control or comparison group such as a waitlist, minimal or alternate intervention, or no-treatment group.

*Outcome:* Studies that reported a health-related outcome (e.g., change in body weight, physical activity, dietary intake) were included.

*Study design:* To ensure the best quality evidence was included in this review, only studies using a RCT or cluster RCT study design were included.

### Search Strategy

Studies reporting on health promotion initiatives delivered in professional sport settings including adult participants were identified through electronic database searches conducted in CINAHL, MEDLINE, SPORTDiscus, Scopus, the Cochrane Central Register of Controlled Trials (CENTRAL), and Google Scholar. The database searches were conducted in August 2019 and an updated search was conducted in July 2021 to ensure all relevant studies were identified and included in this review.

The full search strategy for one database (MEDLINE) is included in ESM Appendix S2. Filters were applied to only include human studies and those published in English, but no date limits were applied. When more than one article reported on the same intervention and the same sample, only the article reporting primary study outcomes was included. Articles reporting on the same intervention but including unique study samples (e.g., a pilot study and a fully powered RCT) were included as separate papers.

### Screening

Two authors (EG and AR) independently screened each identified study based on the title and abstract (stage 1), followed by full-text screening (stage 2). In the event of a disagreement related to study inclusion or reason for exclusion, consensus was reached through discussion with a third author (AE). Reference lists of all included studies were reviewed for additional studies that potentially met the inclusion criteria.

### Data Extraction and Synthesis

Data extracted from eligible studies included authors, year of publication, sporting context, study location, study aims, intervention duration and data collection points, intervention details (including comparison/control group condition and frequency of intervention sessions), study incentives, target population (sex, age, and retention rates), outcome measures, and significant changes in study outcomes. Characteristics of the included studies are reported in Table 1. If a study reported results of per protocol and intention-to-treat (ITT) analyses, ITT results demonstrating a significant difference between groups over time were reported. A qualitative synthesis of intervention elements, characteristics of target populations, and study outcomes are reported.

**Table 1:** Characteristics of the included studies

Authors, sport and study location	Intervention aims, duration and data collection points	Study design and intervention details	Participants	Outcome measures	Main findings (significant, between-groups only)
Gray et al., 2013 [8] Football Fans in Training Pilot (p-FFIT) Football (soccer) Scotland	*Aim:* To evaluate the feasibility and acceptability of FFIT and to explore the potential of FFIT for weight loss, lifestyle and psychological measures *Duration:* 12 weeks *Data collection:* Baseline 12 weeks 6 months (intervention only) 12 months (intervention only)	*Design:* Two-arm pragmatic pilot RCT *Intervention (p-FFIT):* 12-week gender-sensitised weight loss and healthy living programme designed for male football fans. 1 × 90 min intervention session/week at respective club’s home stadium. Weekly sessions comprised classroom-based education on weight management, healthy eating, alcohol consumption and PA, and participants were taught behavioural change techniques (e.g., self-monitoring, goal setting, implementation intentions, feedback on behaviour). The practical PA component involved in-stadia, coach-led PA (including walking, cardiovascular, strength and flexibility exercises, and small-sided football games), and a pedometer-based walking programme. All participants received a standard information booklet on weight loss upon enrolment. Club-based incentives included T-shirts in club colours. Men were offered a £20 football club shop voucher for attending follow-up measurement sessions/focus group discussions *Comparison:* Waitlist control, participated in FFIT after a 4-month delay. Received a standard information booklet on weight loss upon enrolment (same as intervention)	*Participants:* 103 men aged 35–65 years (mean age = 47.1 ± 8.4 years) with a BMI of ≥ 27 kg/m^2^ across two Scottish Premier League football clubs Intervention (*n* = 51), mean age = 48.2 ± 8.4 years, mean BMI = 34.5 ± 3.9 kg/m^2^ Comparison (*n* = 52), mean age = 45.9 ± 8.4 years, mean BMI = 34.5 ± 6.0 kg/m^2^ *Retention:* Intervention 12 weeks: 86.3%, 6 months: 80.4%, 12 months: 78.4% Comparison 12 weeks: 80.8%	*Primary outcome:* Feasibility and acceptability (recruitment, randomisation, data collection and retention) *Secondary outcomes:* Weight (kilograms and percentage change) WC Systolic and diastolic BP Body fat (percentage change) PA and sitting time (self-reported, IPAQ short form) Diet (self-reported, adapted DINE) Alcohol consumption (self-reported, 7-day recall) Psychological health (self-reported; self-esteem, RSE scale; positive and negative affect, PANAS short form) QoL (mental and physical, SF-12)	Significant differences (*p* ≤ 0.05) in p-FFIT vs. waitlist at 12 weeks in: ↓ weight (kg) ↓ Percentage weight loss ↓ WC ↓ Systolic BP ↓ Body fat percentage ↑ Total activity (MET min/week) ↑ Vigorous activity (MET min/week) ↑ Moderate activity (MET min/week) ↑ Breakfast consumption (times/week) ↓ Bacon/processed meats (times/week) ↓ Crisps ↑ Fruit and vegetables (times/day) ↓ Chocolates/sweets (times/day) ↓ Biscuits (times/day) ↓ Sugary drinks (times/day) ↑ Self-esteem
Hunt et al., 2014 (RCT) [10] Gray et al., 2018 (3.5-year follow-up) [29] Football Fans in Training (FFIT) Football (soccer) Scotland	*Aim:* To assess the effectiveness of FFIT on body weight in male football (soccer) fans *Duration:* 12 weeks *Data collection:* Baseline 12 weeks 12 months 3.5 years	*Design:* Two-group pragmatic RCT *Intervention (FFIT):* Same classroom-based content and PA sessions as Gray et al. [8]. All participants received a standard British Heart Foundation booklet on weight management at baseline. Club-based incentives included T-shirts in club colours. Participants who attended the 12-month measurements were offered a £40 club voucher 12-week active (intervention) phase followed by 6 e-mail prompts over 9 months and group reunion at 6 months post-intervention *Comparison:* Waitlist control, received intervention 12 months post-baseline. Participants also received a standard British Heart Foundation booklet on weight management at baseline (same as intervention)	*Participants:* 747 men aged 35–65 years (mean age = 47.1 ± 8.0 years) with BMI ≥ 28 kg/m^2^ across 13 Scottish Premier League football clubs Intervention (*n* = 374), mean age = 47.0 ± 8.07 years, mean BMI = 35.5 ± 5.1 kg/m^2^ Comparison (*n* = 373), mean age = 47.2 ± 7.89 years, mean BMI = 35.3 ± 4.9 kg/m^2^ *Retention:* Intervention 12 weeks: 88.2% 12 months: 89.0% Comparison 12 weeks: 92.8% 12 months: 94.9%	*Primary outcome:* Weight (kilograms and percentage change) *Secondary outcomes:* WC BMI Body fat (percentage change) Systolic and diastolic BP PA (self-reported, IPAQ short form) Diet (self-reported, adapted DINE) Alcohol consumption (self-reported, 7-day recall) Psychological health (self-reported; self-esteem, RSE scale; positive and negative affect, PANAS short form) QoL (mental and physical, self-reported, SF-12)	Significant differences (*p* ≤ 0.05) in FFIT vs. waitlist at 12 weeks and 12 months in: ↓ weight (kilograms) ↓ Percentage weight loss ↓ BMI ↓ WC ↓ Body fat percentage ↓ Systolic and diastolic BP ↑ Self-reported total PA (MET min/week) ↓ Fatty food score ↑ Fruit and vegetable score ↓ Sugary food score ↓ Alcohol consumed (units/week) ↑ Self-esteem ↑ Positive affect ↓ Negative affect ↑ Physical health QoL ↑ Mental health QoL (12 weeks only, not maintained at 12 months) Long-term (3.5 year) follow-up (Gray et al., 2018 [29]) results (*n* = 488, 65%) No between-group differences in primary or secondary outcomes, however weight loss was sustained in both the intervention (− 2.90 kg, *p* < 0.001) and waitlist control groups (− 2.71 kg, *p* < 0.001), and changes in PA and diet were also sustained
Kwasnicka et al., 2020 [19] Aussie Fans in Training (Aussie-FIT) Australian Rules football (AFL) Australia	*Aim:* To test the feasibility of delivering and evaluating the preliminary efficacy of Aussie-FIT *Duration:* 12 weeks *Data collection:* Baseline 12 weeks 6 months (maintenance for intervention, ‘post-programme’ assessment for control, based on programme participation at 3 months)	*Design:* Two-group pilot waitlist RCT *Intervention (Aussie-FIT):* 12-week gender-sensitised programme comprising 1 × 90 min session/week with ~ 15 men/group (two groups/club in intervention, two groups/club in control). Sessions held at team training grounds (one professional and one lower-grade) and included classroom-based activities (focused on PA, healthy eating, weight loss, designed to teach strategies for self-regulation, goal setting, avoiding compensatory behaviours, preventing relapse) and practical PA sessions (including aerobic, strength, flexibility activities, AFL drills, small-sided games). PA increased gradually in duration and intensity as the programme progressed, while education session duration reduced. Participants could access intervention summaries via a closed programme website All participants received an Aussie-FIT booklet with session summaries, Fitbit Zip monitor, club T-shirt and reusable ‘LiveLighter’ branded water bottle. Participants also received an AU$$20 team store voucher at the completion of each assessment session Participants and Aussie-FIT coaches were invited to join closed Facebook groups. Automated text messages encouraging attendance and describing upcoming weekly sessions were sent weekly *Comparison:* Waitlist control group, received access to the programme at 3 months (after end of intervention follow-up)	*Participants:* 130 men aged 35–65 years (mean age: 45.8 ± 7.9 years) with a BMI of ≥ 28 kg/m^2^, across two AFL clubs in Western Australia Intervention (*n* = 64), mean age = 44.2 ± 7.6yrs, mean BMI = 34.7 ± 4.63 kg/m^2^ Comparison (*n* = 66), mean age = 47.2 ± 8.0 years, mean BMI = 35.3 ± 6.53 kg/m^2^ *Retention:* Intervention 12 weeks: 78.0% 6 months: 54.0% Comparison: 12 weeks: 93.0% 6 months: 71.0%	*Primary outcome:* Weight (kilograms and percentage change) *Secondary outcomes:* WC BMI Systolic and diastolic BP PA and sedentary time (ActiGraph GTX-9 accelerometer) Diet (self-reported, Australian adaptation of DINE questionnaire) Alcohol consumption (self-reported, 7-day recall) Wellbeing (self-reported; self-esteem, RSE scale; positive and negative affect, PANAS short form) Perceptions of psychological need support for weight loss (self-reported, IBQ) Motivation for weight loss (self-reported, adapted Treatment Self-Regulation Questionnaire of weight loss motivation) Health-related QoL (self-reported, EQ-5D-5L visual analogue scale) Goal facilitation and competing goals for weight loss goals, barriers, planning, and habits for PA and healthy eating (self-reported, SRBAI) Sleep (self-reported, PSQI) Feasibility (recruitment and retention)	Significant differences (*p* ≤ 0.05) in Aussie-FIT vs. waitlist at 12 weeks in: ↓ Weight (kilograms) ↓ Percentage weight lost ↓ BMI ↑ MVPA (mins/day) ↓ Fatty food score ↓ Sugary food score ↑ Self-esteem ↑ Positive affect ↑ Basic need satisfaction in relation to weight loss ↑ Overall health ↑ Goal facilitation ↑ Habits for PA ↑ Habits for eating ↑ Planning ↑ Sleep quality
Maddison et al., 2019 [18] Rugby Fans in Training (RUFIT-NZ) Rugby New Zealand	*Aim:* To investigate the effects of a healthy lifestyle programme (RUFIT-NZ) on weight loss in overweight or obese men *Duration:* 12 weeks Data collection: Baseline 12 weeks	*Design:* Two-arm parallel design RCT *Intervention (RUFIT-NZ):* 12-week gender-sensitised weight loss programme, inspired by FFIT. Delivered across two clubs (Auckland, Dunedin). Auckland: 2 × 90 min sessions/week (1 × weekend (30 min education and 60 min PA), 1 × weekday (90 min PA only); Dunedin: 1 × 120–150 min session/week (60 min PA and 60–90 min education) Sessions delivered at respective clubs by RUFIT-NZ coaches, local health experts and club staff (dietitian and doctor). Standardised educational content delivered across both clubs, focused on PA, nutrition, sleep and sedentary behaviour, SMART goal setting and behaviour change strategies PA sessions: progressive, coach-led PA. Weeks 1–4: aerobic off-feet conditioning, body weight exercises; weeks 5–8: same exercises, introduction of external loads plus running; weeks 9–12: strength, aerobic and anaerobic conditioning. Small-sided rugby games held in Auckland only Control: Waitlist, offered 12-week RUFIT-NZ intervention post 12-week follow-up	*Participants:* 96 men aged 25–65 years with a BMI of ≥ 25 kg/m^2^ and not meeting NZ PA guidelines, across two professional (Super 18) rugby clubs Intervention (*n* = 49^a^), mean age = 40.6 ± 8.9 years Control (*n* = 47^a^), mean age = 44.7 ± 8.9 years *Retention:* Intervention 12 weeks: 75.5% Comparison 12 weeks: 91.5%	*Primary outcome:* Weight (kilograms) *Secondary outcomes:* WC Body fat (percentage change) Resting HR Systolic and diastolic BP Cardiorespiratory fitness (4-km cycle test) Adherence to health guidelines (self-reported composite health behaviour score on smoking, PA [Godin Leisure Time PA Questionnaire], alcohol intake [AUDITC], fruit and vegetable intake [NZ Health Survey]) Feasibility (recruitment and retention)	Significant differences (*p* ≤ 0.05) in RUFIT-NZ vs. waitlist at 12 weeks in: ↓ WC ↓ Resting HR ↓ Diastolic BP ↑ Proportion adherent to three or more health guidelines
Petrella et al., 2017 [17] Hockey Fans in Training (Hockey FIT) Ice hockey Canada	*Aim:* To examine the feasibility of recruiting and retaining men in a 12-week weight loss and healthy lifestyle programme, and the impact of this programme on weight loss *Duration:* 12 weeks *Data collection:* Baseline 12 weeks 12 months (intervention only)	*Design:* Two-arm pilot pragmatic RCT *Intervention (Hockey FIT):* 12-week gender-sensitised weight loss and healthy lifestyle programme delivered at hockey team’s arena and an affiliated health club facility. Active phase: 1 × 90 min session/week, comprising classroom-based education (behaviour change techniques and information sharing on PA and nutrition, to promote mutual learning) and exercise (aerobic, strength and flexibility exercises practiced ‘off the ice’). Personalised target HR assessed and provided at two time points. Weekly sessions supplemented with an incremental pedometer-based walking programme, and PA/nutrition tracking via the Health*e*Steps smartphone app Participants received a Hockey FIT branded shirt and puck, branded merchandise from the Hockey clubs, free tickets to a match if they attended the reunion/booster, and a $20 gift card to a local sporting store if they attended 12-month measurements Post-intervention, 40-week minimally supported phase: ongoing PA tracking (vie Health*e*Steps app), Hockey FIT social network, six standardised messages via app/e-mail, group reunion and 60-min booster session at 9 months *Comparison:* Waitlist control, received programme approximately 4 months post-baseline after 12-week measurements	*Participants:* 80 men aged 35–65 yrs (mean age = 48.7 ± 9.0 years), with a BMI of ≥ 28 kg/m^2^, across two Ontario Hockey League teams, Canada Intervention (*n* = 40), mean age = 49.1 ± 9.1 years, mean BMI = 36.0 ± 5.9 kg/m^2^ Comparison (*n* = 40), mean age = 48.4 ± 9.1 years, mean BMI = 37.1 ± 6.1 kg/m^2^ *Retention:* Intervention 12 weeks: 82.5% 12 months: > 75.0% Comparison 12 weeks: 85.0%	*Primary outcome:* Feasibility (recruitment and retention) *Secondary outcomes:* Weight (kilograms and percentage change) BMI WC Systolic and diastolic BP PA (Yamax Digiwalker SW-200 pedometer and self-reported, IPAQ short form) Sitting time (self-reported, IPAQ short form) Healthful eating score (self-reported, Starting the Conversation questionnaire) Diet (self-reported, adapted DINE) Alcohol consumption (self-reported, 7-day recall) Psychological health (self-reported; self-esteem, RSE scale; positive and negative affect, PANAS short form) Self-rated health (self-reported, EQ-5D-3L visual analogue scale score)	Significant differences (*p* ≤ 0.05) in Hockey FIT vs. waitlist at 12 weeks in: ↓ Weight (kilograms) ↓ Percentage weight lost ↓ BMI ↓ WC ↓ Systolic BP ↑ Average steps/day (pedometer measured) ↑ Healthful eating score ↓ Fatty food score ↑ Self-rated health ↑ Fruit and vegetable consumption (≥ 3 times/day)
Wyke et al., 2019 [14] European Fans in Training (EuroFIT) Football (soccer) England, The Netherlands, Norway, Portugal	*Aim:* To evaluate the effectiveness of EuroFIT to improve physical activity and sedentary time in male football fans *Duration:* 12 weeks *Data collection:* Baseline 12 weeks 12 months	*Design:* Pragmatic two-arm RCT *Intervention (EuroFIT):* 12-week group-based programme delivered by coaches in football club stadia. 1 × 90 min session/week with 15–20 men/session, combining interactive learning of behaviour change techniques with graded group-based PA. Trained coaches taught participants to select behaviour change techniques from a ‘toolbox’ and emphasised the benefits of becoming more active, sitting less and consuming a healthier diet. Practical PA sessions were progressive and coaches were instructed to emphasise the importance of warm-up activities for injury prevention. All participants received a EuroFIT training shirt and a manual including self-monitoring forms. A pocket-worn, validated device (SitFIT) was also provided to monitor sedentary and non-sedentary time (time spent upright) in addition to daily steps. Participants were trained on the use of the SitFIT device and were encouraged to track progress against individualised, incremental goals to increase step count and time spent upright. The MatchFIT web/smartphone app was designed to track SitFIT data, to play team games and to communicate with other participants. Between-session and post-programme peer support was encouraged via the MatchFIT app or social media platforms *Comparison:* Waitlist control, received access to the programme 12 months post-baseline	*Participants:* 1113 men aged 30–65 years with a BMI of ≥ 27 kg/m^2^ across 15 professional football clubs in England and Europe Intervention (*n* = 560), mean age = 45.9 ± 9.0 years, mean BMI = 33.1 ± 4.6 kg/m^2^ Control (*n* = 553), mean age = 45.6 ± 8.7 years, mean BMI = 33.4 ± 4.7 kg/m^2^ *Retention:* Intervention 12 weeks: 91.0% (83.0% with valid data on main outcome) 12 months: 88.0% (81.0% valid data) Control 12 weeks: 92.0% (85.0% valid data) 12 months: 92.0% (85.0% valid data)	Primary outcomes: Total PA (steps/day, objectively measured [activPAL]) Total sedentary time (mins/day, objectively measured [activPAL]) *Secondary outcomes:* Frequency of physically active choices (self-reported, Activity Choice Index) Diet (self-reported, adapted DINE) Alcohol consumption (self-reported, 7-day recall) PA (self-reported, IPAQ short form) Sedentary time (self-reported, Marshall questionnaire) Weight BMI WC Resting BP Fasting glucose Fasting insulin Total cholesterol HDL cholesterol Triglycerides GGT AST ALT HbA1c Insulin immunoassays HOMA_IR_ Wellbeing (self-reported, Cantril ladder) Self-esteem (self-reported, RSE scale) Vitality (self-reported, subjective vitality scale) Health-related QoL (EQ-5D-5L)	Significant differences (*p* < 0.025 for primary outcomes, *p* < 0.05 secondary outcomes) in EuroFIT vs. waitlist at 12 weeks and 12 months in: ↑ Steps/day ↓ Sedentary time (mins/day, 12 weeks only) ↑ Stepping time (mins/day) ↑ Upright time (12 weeks only) ↓ Weight (kilograms) ↓ BMI ↓ WC ↓ Proportion of participants with BMI ≥ 30 kg/m^2^ ↑ Total PA (IPAQ, MET min/week) ↑ Meeting PA guidelines (IPAQ) ↓ Sitting time (Marshall, hours/day) ↑ Activity Choice Index ↓ Fatty food score ↓ Sugary food score ↑ Fruit and vegetable score ↓ Alcohol consumption (12 months only) ↓ Systolic BP (12 months only) ↓ Diastolic BP ↓ Fasting insulin (reported at 12 months only) ↓ HOMA_IR_ (reported at 12 months only) ↓ Triglycerides (reported at 12 months only) ↓ ALT (reported at 12 months only) ↓ GGT (reported at 12 months only) ↑ Wellbeing ↑ Self-esteem ↑ Vitality

*RCT* randomised controlled trial, *PA* physical activity, *BMI* body mass index, *WC* waist circumference, *BP* blood pressure, *DINE* Dietary Instrument for Nutrition Education, *RSE* Rosenberg Self-Esteem scale, *PANAS* Positive and Negative Affect Schedule, *QoL* quality of life, *HR* heart rate, *SF-12* Short Form 12, *EQ-5D-3L* European Quality of Life–5 Dimensions Questionnaire–3 Levels, *EQ-5D-5L* European Quality of Life–5 Dimensions Questionnaire–5 Levels,* MET* metabolic equivalent,* MVPA* moderate-to-vigorous physical activity, *IPAQ* International Physical Activity Questionnaire,  *HDL* high-density lipoprotein, *GGT* gamma-glutamyl transferase, *AST* aspartate aminotransferase, *ALT* alanine aminotransferase, *HbA1c* haemoglobin A1c, *HOMA*_*IR*_ Homeostasis model-estimated insulin resistance, *IBQ* Interpersonal Behaviours Questionnaire, *SRBAI* Self-Report Behavioural Automaticity Index, *PSQI* Pittsburgh Sleep Quality Index, *AUDITC* Alcohol Use Disorders Identification Test Consumption, *app* application, ↑ indicates increased, ↓ indicates decreased

^a^ Only 45 intervention and 39 control participants (87.5% of the reported sample) completed baseline assessments

### Risk of Bias in Individual Studies

Two authors (ESG and AE) independently assessed the risk of bias in the included studies using the revised Cochrane risk-of-bias tool for randomised trials (RoB 2) [23] (ESM Table S1). The RoB 2 assesses risk of bias across five key domains: the randomisation process, deviation from intended interventions, missing outcome data, outcome measurement, and selection of reported results [23]. For each domain, studies were assigned a rating of ‘low risk of bias’, ‘some concerns’, or ‘high risk of bias’. After all five domains had been assessed, an overall risk-of-bias judgement was assigned. Studies assigned ‘low risk’ are those for which low-risk judgements had been assigned for all domains. An overall judgement of ‘some concerns’ was assigned when there were concerns in at least one domain, while an overall judgement of ‘high risk’ was assigned when there was a high risk of bias in at least one domain or where ‘some concerns’ had been identified in multiple domains [23]. Using Cohen’s kappa [24], the interrater reliability for risk-of-bias ratings between the independent reviewers was 0.91, indicating a high level of agreement [25].

### Risk of Bias Across Studies

We examined a funnel plot to investigate risk of publication bias [26]. If smaller studies were found to have larger effects, there would be evidence that publication bias was present in the meta-analysis.

### Data Preparation for Meta-Analysis

All included studies presented data on between-group differences in weight and waist circumference, therefore data on these outcomes were included in the meta-analysis. Authors of all the included studies were contacted to provide any missing data related to sample size, weight or waist circumference, and to verify data we extracted to be used in the meta-analysis. All but one author responded to our data request and we received missing data for four of the six included studies. For the remaining two studies, standard deviations for the change scores for weight and waist circumference were missing. To estimate these missing values, we used the following equation (Eq. 1), which is in line with guidance provided in the Cochrane Handbook [27].

1 $${\mathrm{SD}}_{\mathrm{diff}}=\sqrt{{\mathrm{SD}}_{1}^{2}+{\mathrm{SD}}_{2}^{2}+2r\left({\mathrm{SD}}_{1}\right)\left({\mathrm{SD}}_{2}\right)}$$

### Summary Measures and Synthesis of Results

The differences in change in weight and waist circumference between the intervention and control/comparison conditions from baseline to 12-week follow-up were the summary measures. We used Stata version 16 (StataCorp LLC, College Station, TX, USA) to conduct the meta-analyses using random-effects models. As all studies included in the meta-analyses used a common metric for weight (i.e., kilograms) and waist circumference (i.e., centimetres), non-standardised weighted mean differences were calculated. Variation attributable to heterogeneity was assessed using the I^2^ statistic. If heterogeneity (*I*^2^ > 50%) was found, meta-regression was used to test the impact of potential effect modifiers (i.e., mean baseline age, mean baseline weight/waist circumference). Risk-of-bias score was not investigated as a potential effect modifier due to limited variability.

## Results

**Fig. 1:**
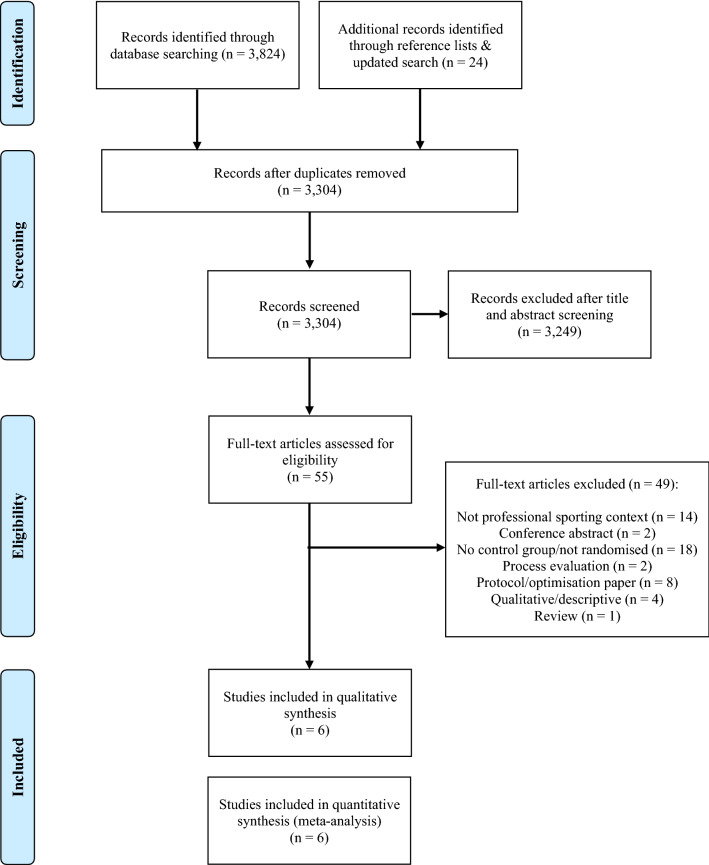
PRISMA flow diagram. *PRISMA* Preferred Reporting Items for Systematic Reviews and Meta-Analyses

Database searches yielded 3824 results. An additional 24 papers were identified through checking reference lists of the included studies and through an updated search in July 2021. After duplicates were removed, 3304 references were screened, with 3249 of these references excluded based on title/abstract, leaving 55 papers for full-text screening. Included in Fig. 1 is the PRISMA flow diagram of study selection, including reasons for exclusion at full-text screening. The most common reasons for exclusion were studies not being delivered in a professional sporting context (*n* = 14) or studies not including a randomised control/comparison group (*n* = 18). A total of six studies reporting on five unique interventions met the inclusion criteria for this review.

### Characteristics of Studies

#### Study Design and Follow-Up Duration

All included studies were either original FFIT studies [8, 10] or an adaptation of the programme [14, 17–19], with varying degrees of modification [28], and all of these studies included members of the original FFIT research team as co-authors. Three studies were delivered via football (soccer) clubs in Scotland [8, 10], Europe and England [14], one through ice hockey teams in Canada [17], one with rugby union in New Zealand [18] and one through Australian Rules football in Australia [19]. All six studies used RCT designs comparing an intervention to a waitlist control group that participated in the intervention after main outcome data were collected [8, 10, 14, 17–19]. Two studies were full-scale trials [10, 14] and four were pilot randomised trials [8, 17–19].

Interventions were conducted across multiple professional sporting clubs in each included study and the number of clubs ranged from two [8, 17–19] to 15 [14]. All interventions in the included studies were 12 weeks in duration and two included an additional maintenance phase ranging from 9 months [10] to 40 weeks [17], with both comprising occasional e-mail prompts and group reunions or ‘booster’ sessions [10, 17]. While all studies assessed intervention effects directly after the intervention (i.e., 12 weeks), five studies conducted longer-term post-baseline follow-up assessments. Two studies assessed between-group differences at 12 months [10, 14], one study conducted 6-month follow-up assessments for intervention participants only [8], one study conducted 6-month follow-up assessments for the intervention group and waitlist control group, which had also completed the intervention by this time [19], and two studies assessed long-term changes at 12 months for intervention participants only [8, 17]. The large-scale FFIT study by Hunt et al. [10] also included a 3.5-year follow-up with 488 men (65% of the original RCT participants) [29].

#### Participant Characteristics, Sample Size and Retention

One study targeted men aged between 25 and 65 years [18], one included those aged 30–65 years [14] and all remaining studies included men aged between 35 and 65 years [8, 10, 17–19]. At baseline, the mean age was 45.83 years in intervention participants, and 46.50 years in control participants, and the mean body mass index (BMI; reported in five studies [8, 10, 14, 17, 19]) was 34.8 kg/m^2^ in intervention participants and 35.1 kg/m^2^ in control participants. Study sample sizes ranged from 80 [17] to 1113 [14]. The two full-scale studies reported conducting a power analysis to detect changes in primary outcomes [10, 14], two pilot studies used a power analysis for a future full-scale trial to inform their pilot sample size [8, 17], and two determined a pragmatic sample size based on guidelines for pilot studies [18, 19]. End-of-intervention retention rates ranged from 75% [18] to 91% [14] in the intervention groups, and from 80% [8] to 93% [19] in the control groups. Of the studies that included a longer follow-up duration of 6 months [19] or 12 months post-intervention [10, 14], retention ranged from 54% [19] to 89% [10] in the intervention groups and 71% [19] to 95% [10] in the waitlist control groups.

#### Intervention Characteristics

The characteristics of the interventions in the included studies are shown in Table 1. Four interventions were delivered wholly at participating clubs’ home stadia [8, 10, 14, 18], one across a club’s training facility and the stadium for the club’s women’s team [19], and one across both the professional club setting (three sessions) and an affiliated health club facility (nine sessions) [17]. Five studies delivered the intervention in weekly 90-min sessions comprising a classroom-based education module and a group-based physical activity component [8, 10, 14, 17, 19]. One study [18] delivered an intervention differently across two clubs. One of these clubs ran the intervention as twice weekly 90-min sessions (one session comprising classroom-based education and group-based physical activity and the other session focused on physical activity only), while the second club delivered one 120- to 150-min session per week, comprising education and physical activity [18]. While most of the included studies were closely modelled on the original FFIT programme [8], the EuroFIT programme incorporated more substantial modifications, including a novel focus on physical activity and sedentary behaviour as desirable health outcomes in their own right [14].

Classroom-based education sessions across programmes focused on weight management [8, 10, 17, 19], healthy eating [8, 10, 14, 17–19], physical activity [8, 10, 14, 17–19], sedentary behaviour [14, 18, 19], sleep [18], and alcohol consumption [8, 10, 17–19], and incorporated behavioural change techniques such as goal setting and self-monitoring [8, 10, 14, 17–19]. Practical physical activity sessions were supervised and group-based in all interventions and involved light- to moderate-intensity activities (involving warm-up/cool down, walking, cardiovascular, strength and flexibility exercises) [8, 10, 17, 19], sport-based drills/training exercises [17, 19], incremental walking programmes [8, 10, 14, 17], physical activities based on individual fitness and ability levels [8, 10, 14, 17–19], and a limited number of small-sided or modified games relevant to the sport in which the intervention was delivered [8, 10, 18, 19].

Self-monitoring devices were utilised in all interventions to assist with monitoring of physical activity, and included pedometers [8, 10, 17, 18], Fitbit Zip devices [19], and a specifically-designed, pocket-worn device (SitFIT) designed to measure sedentary time and step counts [14]. Two interventions included access to a mobile application/online programme enabling self-monitoring. One study utilised a customised mobile application (MatchFIT) designed specifically for the EuroFIT intervention [14] and the other included components from an evidence-based lifestyle prescription programme (Health*e*Steps™) [17].

The group-based intervention design of all studies ensured that an element of social support was embedded within each intervention. Several studies reported additional social support mechanisms including access to mobile applications with social networking capabilities [14, 17] and interaction through commonly used social media platforms such as Facebook and WhatsApp [14, 19].

Intervention delivery personnel varied between studies and included male community coaches from professional clubs [8, 10], male and female graduate kinesiology students with experience in coaching [17], qualified strength and conditioning trainers, club dietitians and medical practitioners, and local health professionals (e.g., nutrition educators) [18], licensed intervention coaches [14], and a combination of coaches from professional clubs and coaches identified by the research team [19]. All studies reported that coaches were trained to provide the intervention, although the detail on coach training varied and one study noted that training was completed by participating sport clubs and did not follow a standardised training protocol [18].

Programme incentives were reported in five studies [8, 10, 14, 17, 19] and included training shirts with intervention branding and/or club colours [8, 10, 14, 17, 19] and Fitbit physical activity trackers [19]. Study incentives such as tickets to matches or merchandise vouchers were also offered for completing follow-up measurements in four studies [8, 10, 17, 19].

#### Intervention Outcomes

Primary outcome measures across included studies were relatively homogenous. Three studies reported weight (kilograms and percentage change) [10, 18, 19] as their primary outcome, two pilot studies assessed feasibility and acceptability as a primary outcome with weight loss assessed as a primary outcome for a definitive trial [8, 17], and one full-scale trial assessed objectively measured physical activity and sedentary time as the primary outcome [14]. Secondary outcomes included waist circumference (*n* = 6) [8, 10, 14, 17–19], systolic and diastolic blood pressure (*n* = 6) [8, 10, 14, 17–19], BMI (*n* = 4) [10, 14, 17, 19], body fat percentage (*n* = 3) [8, 10, 18], weight (*n* = 3) [8, 14, 17], self-reported physical activity (*n* = 4) [8, 10, 14, 17], objectively measured physical activity (*n* = 2) [17, 19], self-reported sedentary time (*n* = 3) [8, 14, 17], objectively measured sedentary time (*n* = 2) [14, 19], dietary intake (*n* = 5) [8, 10, 14, 17, 19], alcohol consumption (*n* = 5) [8, 10, 14, 17, 19], healthful eating score (*n* = 1) [17], self-esteem (*n* = 5) [8, 10, 14, 17, 19], quality of life (*n* = 4) [8, 10, 14, 19], positive and negative affect (*n* = 4) [8, 10, 17, 19], wellbeing (*n* = 1) [14], heart rate (*n* = 1) [18], cardiorespiratory fitness (*n* = 1) [18], self-rated health (*n* = 1) [19], overall health [19], motivation for weight loss (*n* = 1) [19], goal facilitation and competing goals for weight loss, goals, barriers, and planning; and habits for physical activity and healthy eating (*n* = 1) [19], vitality (*n* = 1) [14], sleep (*n* = 1) [19], perceptions of psychological need support for weight loss (*n* = 1) [19], basic need satisfaction in relation to weight loss behaviours (*n* = 1) [19], frequency of physically active choice (*n* = 1) [14], and adherence to health guidelines (*n* = 1) [18]. Two studies assessed the intervention feasibility, including recruitment, randomisation procedures, and participant retention as secondary outcomes [18, 19]. One study [14] collected blood samples to examine additional secondary outcomes, including fasting glucose, fasting insulin, total cholesterol, high-density lipoprotein (HDL) cholesterol, triglycerides, gamma-glutamyl transferase (GGT), aspartate aminotransferase (AST), alanine aminotransferase (ALT), haemoglobin A1c (HbA1c), insulin immunoassays, and homeostasis model-estimated insulin resistance (HOMA_IR_).

#### Study Quality

With regard to risk of bias, four studies were rated as ‘low risk’ [10, 14, 17, 19], one study was rated as ‘some concerns’ [8], and one study was rated as ‘high risk’ of bias [18]. As all included studies tested a health promotion programme against a waitlist control group, there was no way of blinding participants or programme delivery staff to intervention assignment, and therefore this item was not included in the overall assessment-of-bias rating. Concerns regarding outcome assessments were raised in two studies [8, 18], detail on allocation concealment was unclear in one study [8], and bias due to missing outcome data was identified in one study [18]. In this study, participants were randomised prior to baseline measurement sessions and only 87.5% of the reported baseline sample completed baseline measurement sessions [18].

#### Study Results

##### Weight

**Fig. 2:**
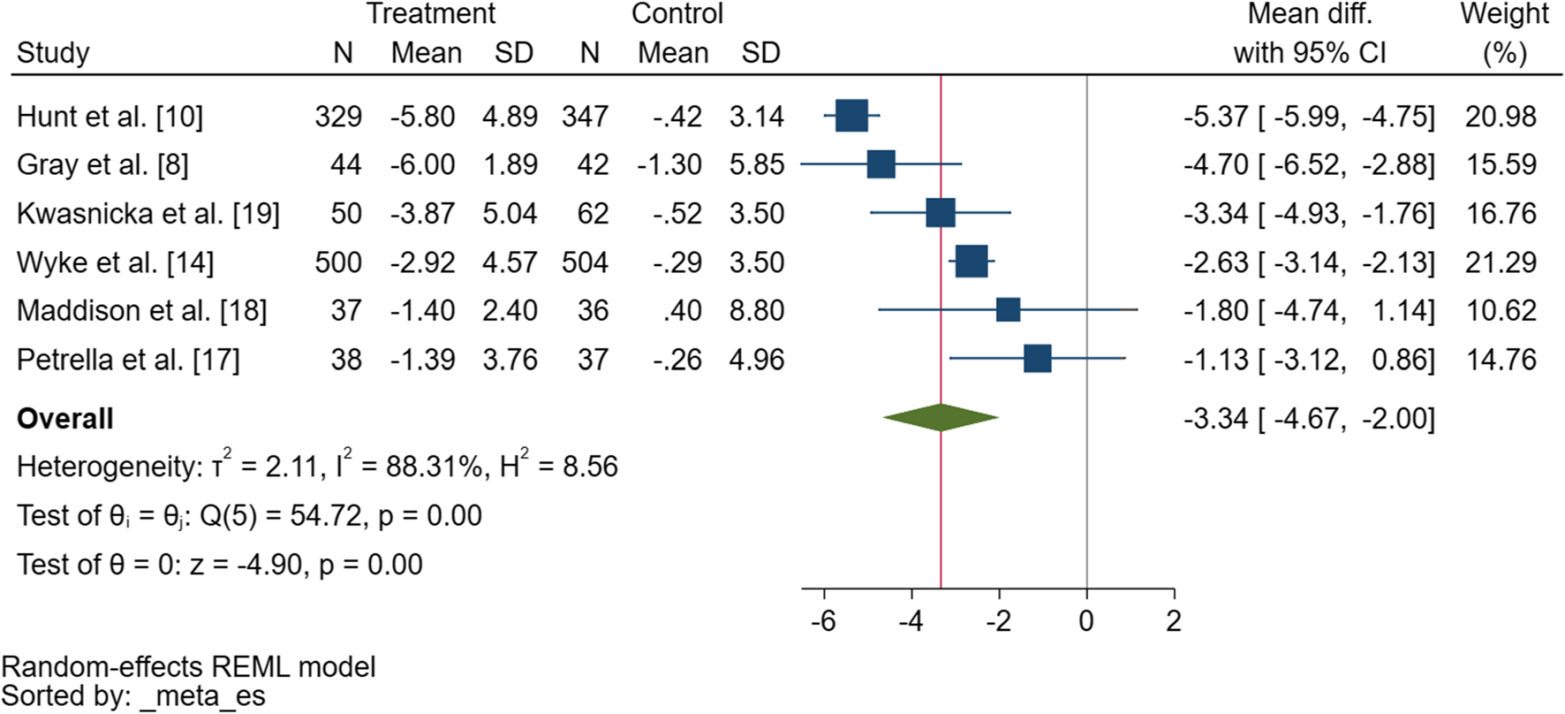
Forest plot showing the difference in change in weight (kilograms) between the intervention and control conditions from baseline to 12-week follow-up. *SD* standard deviation, *CI* confidence interval

Findings from the meta-analysis revealed an overall significant difference in change in weight of − 3.3 kg (95% confidence interval [CI] − 4.7 to − 2.0) in favour of the intervention group at 12 weeks (Fig. 2). Heterogeneity was high (*τ*^2^ = 2.11, *I*^2^ = 88.3%) and no predictors explained this variation (i.e., baseline age, weight). Review of a funnel plot (ESM Fig. S1) and Egger’s test (*p* = 0.31) suggested no evidence of asymmetry or small study bias, indicating no evidence of publication bias.

Five studies reported significant between-group differences in weight directly post-intervention [8, 10, 14, 17, 19], with between-group differences sustained in two studies at 12-month follow-up [10, 14]. All five studies that led to significant changes in weight implemented a standardised programme across multiple clubs and incorporated classroom-based education on behaviour change techniques such as goal setting and self-monitoring. All six studies provided education on physical activity and healthy eating in relation to weight loss and healthy living; however, the study by Wyke et al. [14], which had the greatest level of adaptation from the original FFIT programme, emphasised the importance of increasing physical activity and reducing sedentary time rather than weight loss (which was a secondary outcome). To explore the long-term maintenance of weight loss among participants in the FFIT RCT [10], Gray et al. conducted a 3.5-year longitudinal study with 488 men, representing 65% of all RCT participants. Weight loss was sustained in both intervention (− 2.90 kg; *p* < 0.001) and waitlist control participants (− 2.71 kg; *p* < 0.001) who had also participated in the intervention by this time [29].

Two other studies assessed intervention group weight loss at 6 months [19] and 12 months post-baseline [17] and weight loss was sustained in intervention participants in one study [17]. All four studies that examined differences in the percentage of weight lost reported significant differences between groups post-intervention [8, 10, 17, 19], with weight loss ranging from 3.41% [19] to 5.23% [10].

##### Waist Circumference

**Fig. 3:**
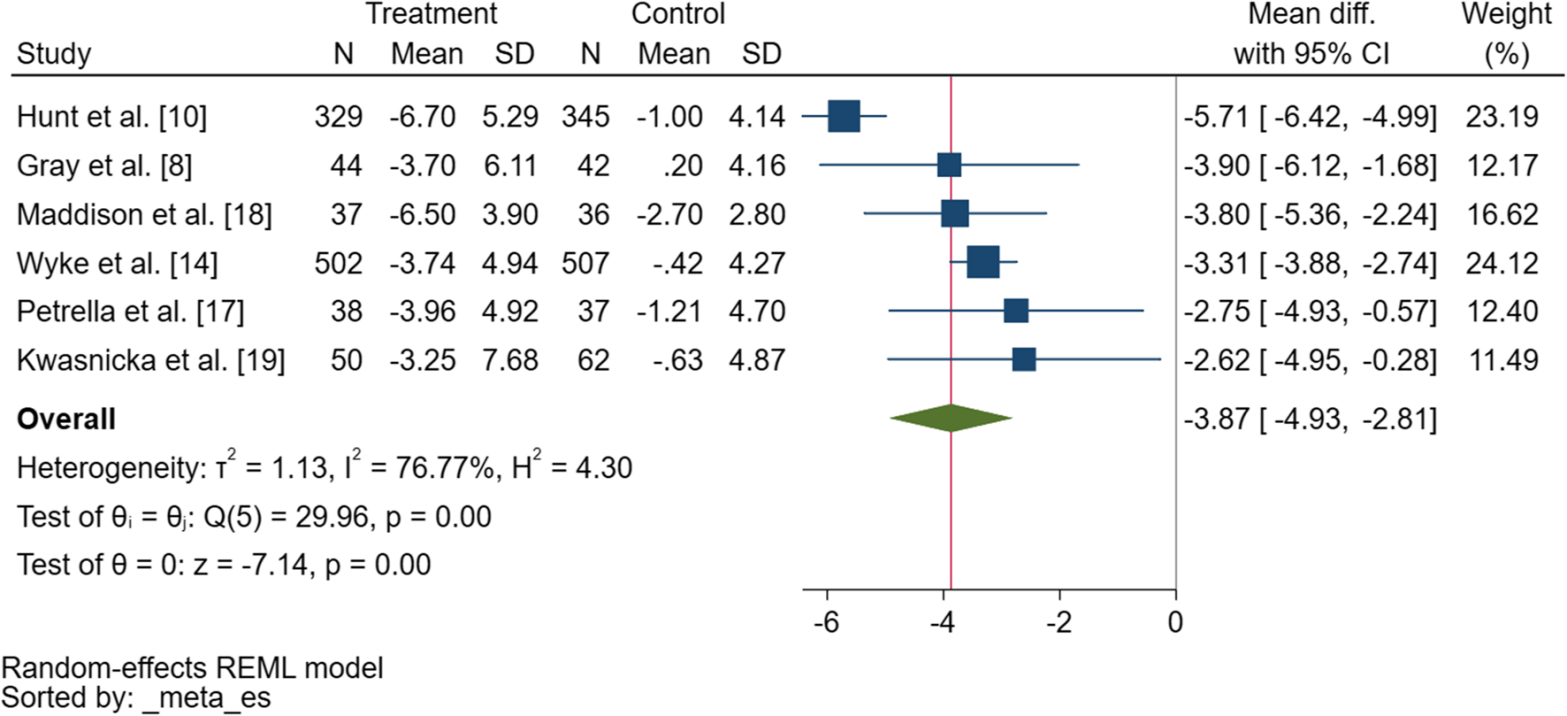
Forest plot showing the difference in change in waist circumference (centimetres) between the intervention and control conditions from baseline to 12-week follow-up. *SD* standard deviation, *CI* confidence interval

Statistically significant between-group differences in waist circumference were reported in five of six studies at 12 weeks [8, 10, 14, 17, 18] and in two studies at 12 months [10, 14]. Findings from the meta-analysis revealed an overall significant difference in change in waist circumference of − 3.9 cm (95% CI − 4.9 to − 2.8) in favour of the intervention group at 12 weeks (Fig. 3). Heterogeneity was high (*τ*^2^ = 1.13, *I*^2^ = 76.8%) and no predictors explained this variation (i.e., baseline age, waist circumference). Visual inspection of a funnel plot (ESM Fig. S2) shows some evidence of asymmetry; however, Egger’s test suggested no evidence of small study bias (*p* = 0.29).

##### Physical Activity and Sedentary Time

Physical activity was objectively measured in three studies via accelerometry [19], ActivPAL devices [14], and pedometers [17], and self-reported in four studies [8, 10, 14, 17]. One study included fitness as a secondary outcome, assessed using a 4 km cycle test [18]. Sedentary time was measured in four studies using accelerometry (*n* = 1) [19], ActivPAL devices (*n* = 1) [14], and self-report methods (*n* = 3) [8, 14, 17]. Five studies reported a significant between-group difference in a physical activity outcome at 12 weeks [8, 10, 14, 17, 19]. Significant between-group differences were observed in overall self-reported physical activity in three studies at 12 weeks [8, 10, 14] and two studies at 12 months [10, 14]. Significant between-group differences were also reported for objectively measured moderate to vigorous physical activity in one study at 12 weeks [19], objectively measured steps per day in two studies at 12 weeks [14, 17] and one study at 12 months [14]. Significant changes in favour of the intervention group were reported in one study [14] for upright time at 12 weeks, stepping time at 12 weeks and 12 months, and frequency of physically active choices at 12 weeks and 12 months. Significant between-group differences were also reported for habits for physical activity in one study at 12 weeks [19]. Of the four studies that measured sedentary time, only one reported significant between-group differences for both objectively measured and self-reported sedentary time at 12 weeks and self-reported sedentary time at 12 months [14]. No other significant changes in sedentary time, either objectively measured or self-reported, were observed.

##### Dietary Intake

Five studies examined changes in dietary intake using an adapted version of the Dietary Instrument for Nutrition Education and a 7-day recall questionnaire to assess alcohol consumption [8, 10, 14, 17, 19], and one reported an overall healthful eating score [17]. Significant between-group differences were reported for fatty food in four studies at 12 weeks [10, 14, 17, 19] and two studies at 12 months [10, 14], fruit and vegetable intake in four studies at 12 weeks [8, 10, 14, 17] and two studies at 12 months [10, 14], and sugary food in three studies at 12 weeks [10, 14, 19] and two studies at 12 months [10, 14]. Significant between-group differences were reported for alcohol consumption in one study at 12 weeks [10] and two studies at 12 months [10, 14], breakfast consumption, bacon and processed meats, crisps, chocolates and sweets, biscuits, and sugary drinks in one study at 12 weeks [8], overall healthful eating score in one study at 12 weeks [17], and habits for eating in one study at 12 weeks [19].

##### Anthropometric Outcomes

All included studies assessed changes in blood pressure and significant between-group differences were reported for diastolic blood pressure at 12 weeks in three studies [10, 14, 18] and 12-month follow-up in two studies [10, 14]. Significant changes in systolic blood pressure were also reported in three studies at 12 weeks [8, 10, 17] and in two studies at 12-month follow-up [10, 14]. Of the three studies that measured body fat as a secondary outcome [8, 10, 18], two studies reported a significant reduction at 12 weeks [8, 10] and one study reported 12-month follow-up [10]. Significant improvements in BMI were reported in all four studies that assessed changes for this outcome at 12 weeks [10, 14, 17, 19] and in two studies at 12-month follow-up [10, 14]. One study reported a significant difference in resting heart rate at 12 weeks [18] and one study that assessed cardiometabolic blood markers reported significant improvements in a range of these markers at 12 months [14].

##### Psychological and General Health-Related Outcomes

A significant increase in self-esteem, assessed using the Rosenberg Self-Esteem Scale, was reported in four studies at 12 weeks [8, 10, 14, 19] and two studies at 12-month follow-up [10, 14]. Two studies reported a significant increase in positive affect at 12 weeks [10, 19], with the increase sustained in one study at 12 months [10], and one reported a decrease in negative affect at 12 weeks and 12-month follow-up [10], assessed using the Positive and Negative Self Affect Schedule. One study reported significant between-group differences in wellbeing and vitality at 12 weeks and 12-month follow-up [14], one reported a significant between-group difference in overall health [19] and one reported a significant between-group difference in self-rated health [17] at 12 weeks. Of the four studies that assessed quality of life [8, 10, 14, 19], one study [10] reported a significant change in physical health-related quality of life at 12 weeks and 12 months, and in mental health-related quality of life at the end of intervention. In one study, significant between-group differences were also reported at 12 weeks for basic need satisfaction in relation to weight loss and goal facilitation for weight loss [19].

##### Sleep

In the one study that measured sleep, sleep quality improved significantly at 12 weeks in favour of the intervention group [19].

## Discussion

### Overview of Findings

This is the first systematic review to explore the effectiveness of health promotion initiatives delivered in a professional sporting context. Meta-analyses revealed small but significant changes in weight and waist circumference in favour of the experimental conditions at 12 weeks. Improvements were also observed for outcomes including physical activity [8, 10, 14, 17, 19], sedentary time [14], dietary intake [8, 10, 14, 17, 19] and a range of psychological and social health outcomes [8, 10, 14, 17, 19].

Sport has long been recognised as an institution in which masculinity is constructed and reinforced [30, 31], and sporting environments such as football clubs have been identified as both opportune spaces to engage men in “constructive reflection on their health and wellbeing” and spaces in which “damaging constructions of gender and inequalities” may be reproduced [32]. In recent years, and as demonstrated through this systematic review, sport has been established as a strong entry point through which to engage men for health promotion [8–10]. This is promising given that, compared with women, men tend to be less engaged with health services and in health promotion and weight loss initiatives [33–35]. All studies that met the inclusion criteria for this systematic review were designed specifically to engage men, with findings suggesting that the professional sporting context is a suitable and accessible setting through which men’s physical and mental health can be promoted and supported. In line with the original aim and design of the FFIT programme, authors of all studies included in this review discussed the ‘gender-sensitised’ nature of their interventions [8, 10, 14, 17–19], which are designed to appeal to masculine ideals, preferences and needs. This gender-sensitised approach seeks to work with, rather than against, traditional constructions of masculinity, and key components of gender sensitisation in these studies include delivery of the intervention within the environment of a sporting club, provision of club-inspired merchandise (e.g., team training shirts), male-only groups, simple health messaging, and the use of humour or ‘banter’ to encourage camaraderie and open discussion [28]. There are many complex sociocultural factors influencing men’s engagement with health behaviours [36, 37] that must be considered in the design of gender-tailored health programs. One of the guiding principles for the development of the original FFIT programme was to enhance men’s “physical and symbolic proximity to the club and fellow male supporters” in an effort to counter potential perceived threats to masculinity [38] and masculine capital [37], which may be associated with involvement in a weight management programme. The successful adaptation and implementation of the FFIT programme across multiple demographic and sporting contexts demonstrates the programme’s success in supporting men to achieve lifestyle change [28].

The one programme that was identified through our database searches that exclusively targeted women used a pre-post-test study design to examine the feasibility of an FFIT adaptation for women [39]. Although this study did not meet the inclusion criteria for this review, as it was not conducted as a RCT, this feasibility study found that FFIT for women was feasible and acceptable and resulted in a mean 2.87 kg weight loss post-intervention. Findings from the mixed-methods feasibility study indicated that the programme appealed to women and was viewed favourably in comparison with other weight management programs and commercial dietary programs, which were often quite restrictive in their approaches. Women appreciated the group nature of the programme and the salience of the physical activity content, suggesting minor changes to enhance the programme for future delivery [39]. These findings suggest that recruiting women through a professional sport setting for the promotion of weight loss and healthy lifestyles may be efficacious and warrants further investigation.

In terms of weight loss, the findings from the current systematic review align with those of several other studies. In their review of male-only weight loss and weight maintenance interventions, Young et al. [40] found a significant difference in weight change favouring interventions in comparison with no-treatment controls. Characteristics of successful interventions included face-to-face intervention delivery in group settings, higher frequency of contact (i.e., ≥ 2.7 contacts per month), and inclusion of a prescribed energy restriction. Similarly, all studies included in the current systematic review were delivered in group settings and included face-to-face contact on a weekly basis. Borek et al. [41] also examined the effectiveness of group-based weight loss interventions promoting physical activity and nutrition. The mean difference in weight loss between intervention and control groups was 3.5 kg at 6 months, 3.4 kg at 12 months, and 2.6 kg at 24 months post-baseline. Findings from moderator analyses indicated that interventions that exclusively targeted men, those that explicitly targeted weight loss, and those that incorporated feedback mechanisms were more effective than those without these characteristics [41]. Importantly, most studies included in these reviews were of low study quality, prompting a call for rigorously designed, high-quality studies promoting weight loss and maintenance [40, 41].

All included studies in this systematic review incorporated practical physical activity sessions as a technique to not only increase physical activity participation but to also build a sense of camaraderie and belonging amongst participants. Although the methods used to assess physical activity varied across studies, the majority of studies demonstrated a significant increase in physical activity at the end of intervention in comparison with a waitlist control [8, 10, 14, 17, 19]. In a recent meta-analysis exploring the effectiveness of behaviour change interventions on men’s physical activity, Sharp et al. [42] found a significant intervention effect of 0.35 (Cohen’s *d*), which is estimated to be equivalent to an increase of approximately 97 min of total physical activity per week, or 980 steps per day. Interventions included in the review by Sharp et al. [42] that were gender-sensitised, underpinned by a theoretical framework, delivered across a period of 12 weeks or less, and involved at least one contact session per week were associated with greater increases in physical activity in comparison with interventions without these characteristics.

Considering the strong psychological connections sport fans form with their team and the strong sense of camaraderie formed between supporters of the same team [5], it is somewhat surprising that none of the included studies directly promoted mental health through their interventions. By nature, all included interventions evoked the sense of belonging and camaraderie offered through sport participation and fandom, and significant between-group differences were observed for a range of psychosocial outcomes [8, 10, 14, 19].

One study that included a specific focus on social connectedness, but did not meet the study design inclusion criteria for the current systematic review, was the HAT TRICK intervention [43]. This study used a pre-post-test study design without a control or comparison group and was delivered through a major junior ice hockey team in Canada. Significant treatment effects were reported for weekly minutes of moderate physical activity (objectively measured), self-reported moderate and vigorous physical activity, and fat scores, but there was no significant change in social connectedness [43]. In a subsequent study exploring the impact of the HAT TRICK programme on men’s mental health, significant positive changes in depression (assessed using the Male Depression Risk Scale) and mental health (assessed using the MH12) [44] were observed.

### Practical Implications

Study samples were relatively homogenous, with limited ethnic diversity and exclusively male samples. Five of the included studies [8, 10, 14, 17, 19] had a predominantly White sample (range 89.6–99.0%), with only one study reporting a sample comprising 37.75% non-White participants [18]. While the exact reason for the limited ethnic diversity in these samples is unclear, employing strategies to engage men from culturally diverse backgrounds will be key for future interventions designed to engage communities via professional sport. Socioeconomic diversity was also limited in some studies. While Hunt et al. [10] and Gray et al. [8] reported success in engaging men from across the socioeconomic spectrum, Kwasnicka et al. [19] and Petrella et al. [17] both noted limited socioeconomic representation and a need for further research on engaging men from diverse socioeconomic and cultural backgrounds.

All studies that were included in this systematic review were offered at no financial cost to participants. Three studies included the results of a cost-effectiveness analysis of the intervention [10, 14, 19]. One was found to be cost effective [10], one potentially cost effective [19], and one not cost effective in the short-term [14], possibly due to a ceiling effect for the quality-of-life measure on which cost effectiveness was based. The FFIT programme has been successfully scaled up and the original programme has since transitioned to a ‘single-licence franchise model’ that ensures programme fidelity, protects against commercialisation, and supports public health [28]. However, not all adaptations of the programme have been successfully scaled up and translated with greater reach. Considering the need for innovative and evidence-based approaches to engage men in health promotion and weight loss initiatives, it is imperative that these programmes are developed with a view to scalability. One potential approach to support long-term programme implementation, in the absence of ongoing funding, is to employ a paid registration model as is used in the MAN v FAT football programme in the UK and Australia [45]. This programme uses competitive sport, rather than the professional sport context, to support weight loss, and findings from a qualitative evaluation of men’s experiences in the programme reinforce the importance of camaraderie and social connectedness [46]. However, it is worth noting that charging a fee to participate in a health promotion programme may limit participation from some population groups [47], thereby amplifying existing socioeconomic inequalities.

### Strengths and Limitations

The strengths of this systematic review include the rigorous study design, focus on the professional sport environment, and use of meta-analysis to assess the effectiveness of included RCTs on weight and waist circumference. These anthropometric outcomes are directly associated with chronic disease risk and are of public health importance [48, 49]. However, the findings of this systematic review should also be viewed in light of potential limitations. First, we only included studies that were published in English and did not search grey literature for studies that may not be published in scientific journals. Second, the results of the meta-analyses indicated high levels of heterogeneity (*τ*^2^ = 2.57, *I*^2^ = 94.2% for weight; and *τ*^2^ = 1.13, *I*^2^ = 76.8% for waist circumference), and none of the hypothesised predictors explained this variation. Third, this review only included RCTs as they are the highest level in the hierarchy of evidence for intervention studies. Although studies using other designs may also provide important evidence on the impact of health promotion initiatives delivered in a professional sporting context, they were not included in this review. Finally, of the studies that met our inclusion criteria, none were conducted in low- or middle-income countries, and ethnic and socioeconomic diversity was limited.

## Conclusion

The findings of this systematic review highlight the potential for professional sport to be utilised as a vehicle for delivering successful health promotion initiatives for men. When designed to meet the needs of local communities and priority population groups, such interventions have the potential to positively influence health and wellbeing within their communities. The limited number of RCTs that met the inclusion criteria for this review emphasises the need for rigorously designed interventions, with standardised intervention approaches, long-term follow-up, and potential for scalability. Future research could explore the effectiveness of health promotion or weight loss interventions for female sport fans and those in low- and middle-income countries, and should include strategies to engage participants from culturally diverse backgrounds and areas of socioeconomic disadvantage.

## Supplementary Information

Below is the link to the electronic supplementary material.

Supplementary file1 (DOCX 10121 kb)

## Declarations

### Availability of data and material

All data generated or analysed during this study are included in the published article.
